# Comparative Analysis of Concentration and Quantification Methods for Antibiotic Resistance Genes and Their Phage-Mediated Dissemination in Treated Wastewater and Biosolids

**DOI:** 10.3390/pathogens14101050

**Published:** 2025-10-18

**Authors:** Irene Falcó, Ana Allende, Francesca Cutrupi, Rosa Aznar, Gloria Sánchez, Pilar Truchado

**Affiliations:** 1 Department of Microbiology and Ecology, University of Valencia, 46100 Valencia, Spain; 2 Research Group on Microbiology and Quality of Fruits and Vegetables (MxQ), Department of Food Science and Technology, CEBAS-CSIC, Campus Universitario de Espinardo, 25, 30100 Murcia, Spain; 3 CIBIO—Department of Cellular, Computational and Integrative Biology, University of Trento, 38123 Trento, Italy; 4 Environmental Virology and Food Safety Lab (VISAFELab), Department of Preservation and Food Safety Technologies, Institute of Agrochemistry and Food Technology, IATA-CSIC, Av. Agustín Escardino 7, 46980 Valencia, Spain

**Keywords:** wastewater, antimicrobial resistance genes, bacteriophages, biosolids, digital PCR

## Abstract

Antimicrobial resistance poses a growing threat to public health, and integrated surveillance strategies across environmental compartments such as treated wastewater and biosolids can substantially improve monitoring efforts. A key challenge is the diversity of available protocols, which complicates comparability for the concentration and detection of antibiotic resistance genes (ARGs), particularly in complex matrices. In this study, we compared two commonly used concentration methods—filtration–centrifugation (FC) and aluminum-based precipitation (AP)—and two detection techniques, quantitative PCR (qPCR) and droplet digital PCR (ddPCR), for the quantification of four clinically relevant ARGs: *tet(A)*, *bla_CTX-M_ group 1*, *qnr*B, and *catI*. Analyses were performed in both secondary treated wastewater and biosolid samples, including their purified bacteriophage-associated DNA fractions. Results showed that the AP method provided higher ARG concentrations than FC, particularly in wastewater samples. ddPCR demonstrated greater sensitivity than qPCR in wastewater, whereas in biosolids, both methods performed similarly, although ddPCR yielded weaker detection. Importantly, ARGs were detected in the phage fraction of both matrices, with ddPCR generally offering higher detection levels. These results provide comparative insights into established methodologies and highlight the value of selecting appropriate protocols based on matrix characteristics and surveillance objectives.

## 1. Introduction

Antimicrobial resistance (AMR) is a growing One Health concern that threatens the effectiveness of current therapies and represents an important challenge to global public health. The rise in multidrug-resistant and pan-resistant bacteria, commonly referred to as “superbugs”, has led to infections that are increasingly difficult or even impossible to treat [1].

While AMR is often linked to regions with high antibiotic use, antibiotic-resistant bacteria (ARBs) and resistance genes (ARGs) have also been widely detected in aquatic environments, underscoring the environmental dimension of this crisis [2].

Wastewater treatment plants (WWTPs) play a critical role in this context, acting as both sinks and potential amplifiers of ARGs. These facilities receive inputs from domestic, industrial, and hospital sources and can serve as hotspots for the selection, concentration, and dissemination of ARBs and ARGs into receiving water bodies [3]. Recognizing this, the European Commission has prioritized the safe reuse of reclaimed water as a pillar of the circular economy [4], and projected climate-driven water scarcity further underscores the importance of effective monitoring strategies for environmental AMR risks.

Reliable monitoring depends heavily on the sensitivity and reproducibility of the analytical methods used to detect and quantify ARGs. Several techniques are available to detect ARGs from environmental samples, such as filtration [5,6], centrifugation [7,8], ultrafiltration, and precipitation [8,9,10]. Each of these methods has distinct advantages and limitations depending on the matrix type and target organisms. For instance, membrane filtration can miss particles of certain sizes, centrifugation may damage cells, and precipitation efficiency varies with reagent chemistry. These factors can significantly influence downstream detection and highlight the need for method comparison under realistic conditions.

Quantitative PCR (qPCR) remains a widely used tool for ARG detection due to its sensitivity and specificity, enabling quantification across a wide range of microbial taxa [11,12,13]. However, its limitations are well recognized. It cannot detect novel ARGs, distinguish between free and intracellular DNA, confirm host viability, or provide absolute quantification without standard curves. Additionally, qPCR performance can be impaired by matrix-associated inhibitors and by the diversity of protocols available for complex environmental samples, which complicates data comparability [4,8]. 

Droplet digital PCR (ddPCR) has emerged as a robust alternative, offering absolute quantification by partitioning samples into thousands of nanoliter-sized droplets [14,15,16,17]. This technology reduces the impact of inhibitors and has demonstrated enhanced sensitivity in detecting low-abundance ARGs in complex matrices, including water and biosolids [18,19]. However, ddPCR is still less widespread in environmental AMR surveillance, and further evaluation is needed to understand its added value over qPCR.

In parallel, recent studies have highlighted the potential role of bacteriophages in AMR dissemination [20,21], although their actual contribution remains under debate [22,23]. Traditionally overlooked, these viruses are now increasingly recognized as potential vectors of horizontal gene transfer and significant contributors to the spread of ARGs in the environment [24,25].

High-frequency transduction events [26,27] and the intrinsic resistance of phages to conventional disinfection processes [28] raise concerns about their role as ARG reservoirs in treated effluents and biosolids.

According to the latest EFSA scientific opinion [29], the highest-priority ARGs for monitoring include those conferring resistance to carbapenems (e.g., *bla_VIM_*, *bla_NDM_*, *bla_OXA_* variants), extended-spectrum cephalosporins (*bla*_CTX-M_, *AmpC*), colistin (*mcr*), methicillin (*mecA*), glycopeptides (*vanA*), and oxazolidinones (*cfr*, *optrA*). These determinants have been detected in multiple environmental sources, particularly wastewater, soil, and manure, with variable prevalence. Resistance genes conferring reduced susceptibility to tetracyclines, β-lactams, quinolones, and phenicols are also of relevance for environmental AMR monitoring. Tetracycline resistance determinants are among the most abundant and persistent ARGs reported in aquatic and soil ecosystems, while β-lactamase-encoding genes comprise several extended-spectrum variants with high clinical and environmental significance. Quinolone resistance genes, often plasmid-borne, play an important role in the dissemination of mobile resistance elements, and phenicol resistance determinants remain common in environmental microbiomes, reflecting the long-term imprint of anthropogenic antibiotic use [30,31,32,33,34,35].

Given this context, our study compares two widely used concentration methods—filtration–centrifugation (FC) and aluminum-based precipitation (AP)—and two detection techniques—qPCR and ddPCR—for quantifying selected ARGs (*tet(A)*, *bla_CTX-M_ group 1*, *qnrB*, and *catI*) in secondary treated wastewater and biosolids, including their purified phage-associated fractions. The selected targets represent four major antibiotic classes of high clinical and environmental relevance (tetracyclines, β-lactams/ESBLs, quinolones, and phenicols), in line with the priority ARG groups highlighted by EFSA [29]. This integrated design provides three advances for environmental AMR surveillance: (i) a controlled, cross-method comparison applied to the same aliquots across two matrices, (ii) inclusion of phage-associated ARG detection in biosolids—a matrix seldom evaluated in this context, and (iii) quantitative assessment of matrix-dependent performance and mitigation strategies (e.g., ddPCR inhibition relief by dilution). These insights aim to guide protocol selection and harmonization in ARG surveillance frameworks.

## 2. Materials and Methods

### 2.1. Sample Collection

On July 2022, 5 different samples of wastewater treated at the secondary stage, named secondary effluent samples (1 L) and biosolids from urban WWTPs located in Valencia (Spain) were collected in sterile polypropylene plastic bottles (VWR, PA, USA). Samples were stored under refrigeration conditions, transported within 2 h to the laboratory and stored at 4 °C until analysis.

### 2.2. Comparison of ARG Concentration Methods in Treated Wastewater

Initially, the performance of two concentration approaches was tested on five secondary treated wastewater samples using a filtration–centrifugation protocol (FC) and an aluminum-based adsorption precipitation method (AP), each performed in duplicate.

For FC, 200 mL of treated wastewater was filtered through 0.45 µm sterile cellulose nitrate filters (MicroFunnel™ Filter Funnel, Pall Corporation, Exton, PA, USA) under vacuum. The filters were deposited in Falcon tubes containing 20 mL of buffered peptone water (2 g/L + 0.1% Tween) and vigorously agitated before being subjected to sonication for 7 min with an ultrasonic wave power density and frequency of 0.01–0.02 w/mL and 45 KHz, respectively. After removing the filters, the samples were centrifuged at 3000× *g* for 10 min and the pellet was resuspended in PBS and then concentrated by centrifugation at 9000× *g* for 10 min. The supernatant was subsequently discarded, and the pellet was resuspended with 1 mL of PBS [36]. For AP, the pH of 200 mL of wastewater was lowered to 6.0. Precipitation was accomplished by combining 1 part of 0.9 N AlCl_3_ per 100 parts of the sample. The solution was then shaken at 150 rpm for 15 min, centrifuged at 1700× *g* for 20 min, and the pellet was reconstituted in 10 mL of 3% beef extract (pH 7.4) and shaken at 150 rpm for 10 min at room temperature (RT). The resultant suspension was centrifuged for 30 min at 1900× *g* and, finally, the pellet was resuspended in 1 mL of PBS (Pérez-Cataluña et al., 2021) [37]. The concentrated samples from both concentration methods were then frozen at −80 °C until the DNA extraction was performed.

### 2.3. Treated Wastewater and Biosolids DNA Extraction

For biosolid samples, 0.1 g of biosolids was resuspended in 900 μL of PBS prior to nucleic acid extraction for PCR analysis. DNA from wastewater concentrates (using FC and AP methods) and biosolids was extracted and purified using the Maxwell RSC Pure Food GMO and Authentication Kit (Promega, Southampton, UK) along with the Maxwell^®^ RSC Instrument (Promega, UK). Briefly, 300 μL of the concentrated water samples or resuspended biosolids were added with 400 μL of cetyltrimethyl ammonium bromide (CTAB) and 40 μL of proteinase K solution, both provided in the kit. The mixture was then incubated at 60 °C for 10 min and centrifuged at 16,000× *g* for 10 min. Then, the supernatant was transferred together with 300 μL of lysis buffer to the loading cartridge. The cartridge was inserted into the Maxwell^®^ RSC Instrument, and the extraction was conducted using the PureFood GMO program. Ultimately, DNA was eluted in 100 μL nuclease-free water. The extraction procedure includes a negative control consisting of nuclease-free water instead of the concentrated sample.

### 2.4. Purification of Phage Particles

Phages were purified as previously described [38] with some modifications. Briefly, 600 µL of wastewater concentrates obtained by the AP concentration method and biosolids suspensions were filtered through 0.22 μm low protein-binding polyethersulfone (PES) membranes (Millex-GP, Merck Millipore, Burlington, MA, USA). The filtrates were treated with chloroform (10% *v*/*v*) and shaken for 5 min at RT. Then, the two-phase mixture was separated by centrifugation at 4000× *g* for 10 min. The collected aqueous phase was treated with 100 U of DNAse I (Sigma-Aldrich, St. Louis, MO, USA) at 37 °C for 1 h using the reaction buffer provided by the manufacturer. DNase I was then inactivated by heating at 75 °C for 5 min. Finally, DNA extraction was performed as described above.

### 2.5. ARGs Quantification Methods

Four different ARGs, named *tet(A)* that confer resistance to tetracycline, *bla_CTX-M_ group 1* to β-lactam, *qnrB* to quinolones, and *catI* to chloramphenicol, and the total bacteria 16S rRNA gene, used as a positive control, were quantified using qPCR and ddPCR. Primers, probes, and qPCR conditions used in this study are listed in Table 1. The primers used in this study were selected from previously validated publications to ensure high specificity (Table 1). Melting curve analyses in our assays showed single, specific peaks with no amplification in negative controls, confirming primer specificity.

Gene copy numbers obtained from both qPCR and ddPCR were expressed as copies per 100 mL of sample for secondary treated wastewater and per gram of dry weight for biosolids. For the phage fraction, results were normalized per 100 mL of starting material. No further normalization to biomass, DNA yield, or 16S rRNA gene abundance was applied, in order to preserve a direct comparison of absolute concentrations recovered by each method.

#### 2.5.1. qPCR Quantification Settings and Reaction Conditions

The presence of the selected ARGs (*tet(A)*, *bla_CTX-M_ group 1*, *qnrB* and *catI*) was assessed by qPCR using the StepOne Real Time PCR System with the qPCR Premix Ex TaqTM kit (Takara Bio Inc., Tokyo, Japan) for *tet(A)* and the QuantStudio 5 Real Time PCR Instrument using KAPA SYBR FAST 2× mastermix kit (Kapa Biosystems, Wilmington, MA, USA) for the rest of the ARGs. Results were analysed with the Applied Biosystems StepOne Instrument program and Quantstudio™ Design & Analysis software version 2.6 (desktop and Thermo Fisher™ Connect, Waltham, MA, USA), respectively. TaqMan hydrolysis probe (Table 1) was used for *tet(A)* gene, while SYBR green was used for *bla_CTX-M_ group 1*, *qnrB,* and *catI* detection. Reaction mixtures had a final volume of 10 μL containing 2.5 μL of the extracted DNA. All samples were run in duplicate. In all cases, a non-template control (NTC) was included using 2.5 μL of DNAse free water instead of the DNA template. Standard curves were prepared by 10-fold serial dilution using known concentrations of genomic DNA isolated from different bacterial strains. The strains used were *Escherichia coli* CIP 103470 from the Pasteur Institute collection (Pasteur Institute, Paris, France), *Klebsiella pneumoniae* 997156 from Granada University (Granada, Spain), *Citrobacter freundii* CIP 106650, and *E. coli* CIP 111633. These strains carried the following genes: *tet(A)*, *bla_CTX-M_ group 1*, *qnrB*, and *catI*, respectively (Supplementary Material Table S1 and Figure S1).

#### 2.5.2. ddPCR Quantification Settings and Reaction Conditions

The specified ARGs were quantified by ddPCR using the QX200 AutoDG droplet digital PCR system (Bio-Rad, Hercules, CA, USA). Reaction mix consisted of 11 μL of 2 × ddPCR EvaGreen Supermix for *catI*, *qnrB* and *bla_CTX-M_ group 1* genes or ddPCR Supermix for probes (No ddUTP) (Bio-Rad) for *tet(A)* gen. Each reaction included forward and reverse primers at a final concentration of 0.14 µM each, probe at a final concentration of 200 nM, 1.1 μL of extracted DNA, and DNAse-free water to a total volume of 22 μL. After a 15 min cooling period at 4 °C, samples were transferred to a QX200 droplet reader (Bio-Rad). Data acquisition and analyses were performed using the QX Manager Software (version 1.1) (Bio-Rad). All the reactions resulted in more than 10,000 droplets in this study. Two positive and two negative control samples were included in each run. The threshold of each analysis was manually determined for each plate based on data from positive and negative control samples. Each sample was analyzed in duplicate and considered positive if at least 2 wells were positive. In some biosolid samples, mild inhibition was observed and addressed by diluting the DNA extract 1:1000 prior to quantification. This dilution level was selected after verifying proper droplet separation and consistent replicate signals.

##### Statistical Analyses

All data were compiled from independent secondary-treated wastewater and biosolid samples, with at least two technical replicates for each variable. For statistical comparisons, gene copy numbers were log10-transformed prior to analysis to normalize distributions and reduce heteroscedasticity. For comparisons involving two groups (e.g., AP vs. FC or qPCR vs. ddPCR) for each single gene, a Student’s *t*-test was performed on the log-transformed biological replicate means. A two-way analysis of variance (ANOVA) was conducted to assess the effects of both gene type and detection method (qPCR, ddPCR, and diluted ddPCR). Additionally, a two-way ANOVA was used to evaluate the effects of detection method (qPCR vs. ddPCR) and concentration method (AP vs. FC) on ARG detection for the same gene. This analysis also allowed for the evaluation of potential interactions between the detection and concentration methods. Statistical analyses were performed using STATISTICA software, version 7 (StatSoft Inc., Tulsa, OK, USA). Differences were considered significant when *p* < 0.05. Graphs were created using Microsoft Excel for Windows 11.

## 3. Results

### 3.1. Comparison of ARG Concentration Methods in Treated Wastewater by qPCR

**Figure 1:**
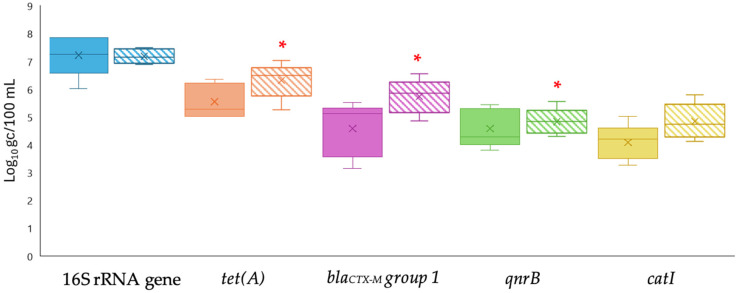
Levels of ARGs measured by qPCR using two concentration methods: filtration–centrifugation (FC, solid colors) and aluminum-based adsorption precipitation (AP, dashed colors) in secondary effluent samples. Targets include the 16S rRNA gene and specific ARGs (*tet(A)*, *bla_CTX-M_ group* 1, *qnrB*, and *catI*). Box plots show the median (line), interquartile range (box), and minimum–maximum values (whiskers). * Asterisks indicate significant differences between FC and AP (*p* < 0.05).

Wastewater samples treated at the secondary stage, named secondary effluent samples, were processed using both the FC and the AP methods to evaluate their ability to concentrate ARGs. Overall, the concentration method based on aluminum precipitation (AP), detected by qPCR, was the most effective, showing significant differences for most of the analyzed ARGs (Figure 1).

Using the AP method, levels of *tet(A)*, *bla_CTX-M_ group 1*, *qnrB*, and *catI* genes were 6.10 ± 0.35, 5.74 ± 0.61, 4.83 ± 0.27, and 4.84 ± 0.43 log gc/100 mL, respectively, while after applying the FC method, the *tet(A)*, *bla_CTX-M_ group 1*, *qnrB*, and *catI* genes showed average levels of 5.57 ± 0.39, 4.50 ± 0.72, 4.57 ± 0.56, and 4.08 ± 0.10 log gc/100 mL, respectively. For the 16S rRNA gene, the FC method yielded a mean gene measurement of 7.22 ± 0.19 log gc/100 mL, whereas the AP method resulted in a mean value of 7.08 ± 0.59 log gc/100 mL, with minimal variation between samples.

### 3.2. Comparison of qPCR and ddPCR Methods for ARG Quantification

**Figure 2:**
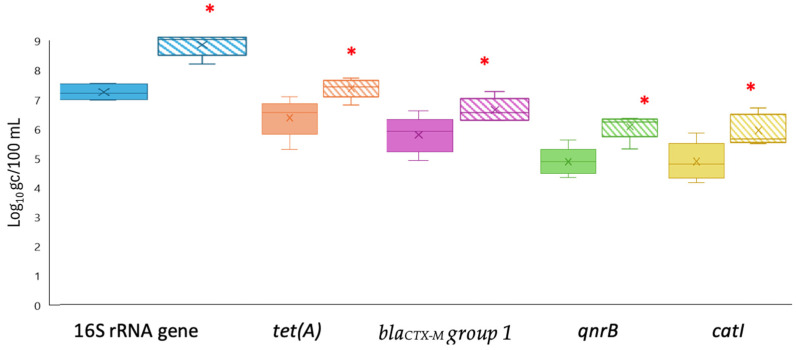
Levels of ARGs measured by ddPCR using two concentration methods: filtration–centrifugation (FC, solid colors) and aluminum-based adsorption precipitation (AP, dashed colors) in secondary effluent samples. Targets include the 16S rRNA gene and specific ARGs (*tet(A)*, *bla_CTX-M_ group* 1, *qnrB*, and *catI*). Box plots show the median (line), interquartile range (box), and minimum–maximum values (whiskers). * Asterisks indicate significant differences between FC and AP (*p* < 0.05).

The levels of four different ARGs (*tet(A)*, *bla_CTX-M_ group 1*, *qnrB*, and *catI*) as well as total bacteria (16S rRNA gene) were determined using qPCR and ddPCR techniques. This comparison was performed on concentrated secondary treated wastewater samples obtained using both FC and AP procedures, as well as on biosolid samples. Overall, ddPCR demonstrated higher sensitivity for the 16S rRNA gene, *tet(A)*, *qnrB*, *bla_CTX-M_ group 1* and *catI* genes in wastewater samples, with average values of 8.75 ± 0.27, 7.39 ± 0.20, 6.58 ± 0.22, 6.00 ± 0.24 and 5.88 ± 0.25 for the AP method and 7.18 ± 0.30, 6.43 ± 0.34, 4.98 ± 0.15, 4.90 ± 0.44 and 4.16 ± 0.31 for FC log gc/100 mL, respectively (Figure 2). In contrast, the use of qPCR reported mean values for both concentration methods of 7.35 ± 0.18, 5.84 ± 0.37, 5.12 ± 0.88, 4.70 ± 0.18 and 4.46 ± 0.54 log gc/100 mL for the same genes (16S rRNA gene, *tet(A)*, *qnrB*, and *catI*), respectively. All wastewater samples concentrated using the AP method and detected using ddPCR exhibited significant differences compared to qPCR (Figure S2). The disparities in quantification for the *tet(A)*, *qnrB*, and *catI* genes were greater than 1 log when detected by ddPCR and concentrated by the AP method, but *bla_CTX-M_ group 1* exhibited a difference of 0.84 log compared to qPCR. However, the FC method only demonstrated significant differences in concentration for the *tet(A)* gene, as its detection was 0.83 log higher by ddPCR than by qPCR.

**Figure 3:**
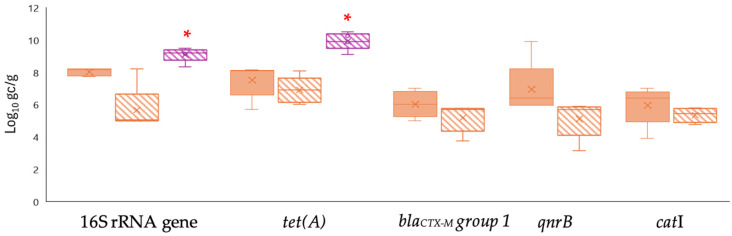
Levels of ARGs quantified by qPCR (solid colors) and ddPCR (dashed colors) in biosolid samples. Targets include the 16S rRNA gene and specific ARGs (*tet(A)*, *bla_CTX-M_ group 1*, *qnrB*, and *catI*). Box plots show the median (line), interquartile range (box), and minimum–maximum values (whiskers). * Asterisks indicate significant differences between FC and AP (*p* < 0.05).

The qPCR and ddPCR were also evaluated for their relative consistency in quantifying ARGs in biosolids samples (Figure 3). In the analysis of biosolids samples, contrary trends were observed since the concentration of *tet(A)*, *bla_CTX-M_ group 1*, *qnrB* and *catI* genes (7.25 ± 0.36, 6.10 ± 0.05, 6.57 ± 0.29 and 5.45 ± 0.23 log gc/g, respectively) were significantly higher (*p* < 0.05) when using qPCR compared to ddPCR. Notably, qPCR outperformed ddPCR in quantifying the total bacteria levels, with an average concentration of 7.64 ± 0.03 log gc/g, significantly higher than the 5.96 ± 0.45 log gc/g reported by ddPCR. Furthermore, specific biosolids samples were 1000-fold diluted, which enhanced ddPCR quantification for the two genes analyzed, resulting in increases of 2.50 and 3 log units.

### 3.3. Detection of ARGs in the Phage Fraction in Treated Wastewater and Biosolids

**Figure 4:**
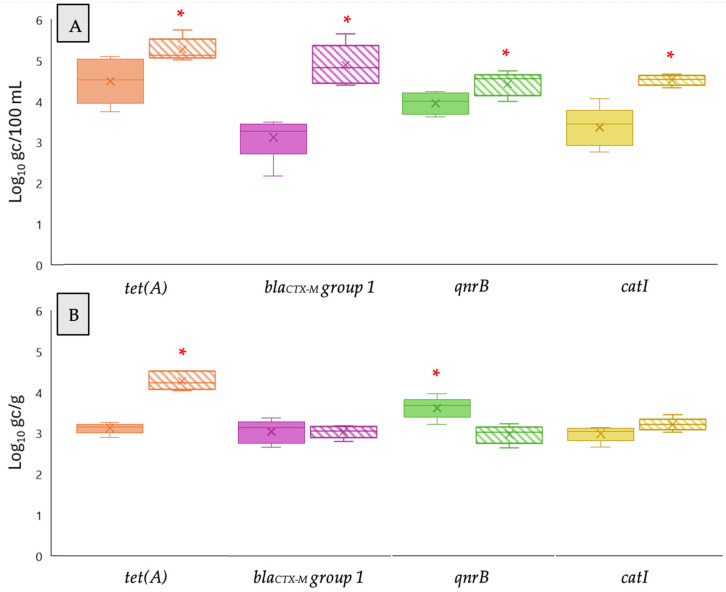
Levels of ARGs detected in the phage fraction of secondary effluent (**A**) and biosolid (**B**) samples using qPCR (solid colors) and ddPCR (dashed colors) after aluminum-based adsorption precipitation (AP). Targets include the 16S rRNA gene and specific ARGs (*tet(A)*, *bla_CTX-M_ group* 1, *qnrB*, and *catI*). Box plots show the median (line), interquartile range (box), and minimum–maximum values (whiskers). * Asterisks indicate significant differences between FC and AP (*p* < 0.05).

ARGs in the purified phage fraction of secondary effluent samples concentrated by the AP method and biosolids were quantified by qPCR and ddPCR. The mean concentration in the phage fraction from treated wastewater by ddPCR was consistently higher than qPCR (Figure 4). Concretely, the concentration of *tet(A)*, *bla_CTX-M_ group 1*, *qnrB*, and *catI* genes was 5.01 ± 0.17, 5.09 ± 0.26, 4.74 ± 0.23, and 4.33 ± 0.13 log gc/100 mL by ddPCR, respectively, in contrast to 4.15 ± 0.36, 3.26 ± 0.29, 3.74 ± 0.18, and 3.08 ± 0.26 log gc/100 mL by qPCR.

When comparing ddPCR and qPCR results on biosolids samples, the average concentration of the *tet(A)* gene was 3.14 ± 0.15 log gc/g for qPCR and 4.04 ± 0.24 log gc/g for ddPCR. The concentration of the *qnrB* gene determined by qPCR was 3.67 ± 0.27 log gc/g, showing significant differences with respect to ddPCR, which reported a slightly lower result, 3.03 ± 0.31 log gc/g. Furthermore, the concentration of the *bla_CTX-M_ group 1* gene measured by qPCR and ddPCR was comparable, with levels of 3.15 ± 0.21 log gc/g and 3.22 ± 0.09 log gc/g, respectively. Similarly, the levels of the catI gene were found to be 3.14 ± 0.19 log gc/g using qPCR and 3.04 ± 0.12 log gc/g using ddPCR. Control samples, including phage DNA extractions, performed as expected and did not show any detection of the 16S rRNA gene.

## 4. Discussion

This study compared two concentration methods (FC and AP) and two detection techniques (qPCR and ddPCR) to quantify *tet(A)*, *bla_CTX-M_ group 1*, *qnrB*, *catI*, and 16S rRNA in secondary treated wastewater and biosolids. All targets were detected across matrices, with *tet(A)* most abundant and *qnrB* and *catI* least represented, consistent with prior work [36,44]. Among the genetic targets studied, the *tet(A)* gene showed the highest abundance, while *qnrB* and *catI* were the least represented. Notably, *bla_CTX-M_ group 1*_,_ a clinically relevant gene encoding extended-spectrum β-lactamases (ESBLs), frequently found in *E. coli* and *K. pneumoniae*, was detected at levels slightly above those reported in earlier studies [45,46]. These bacteria are classified by the World Health Organization as critical priority pathogens due to their multidrug resistance and clinical impact [29]. These results highlight the value of sensitive and reliable methods for environmental AMR monitoring.

Secondary treated wastewater was selected as an important intermediate stage where ARGs and bacteriophages remain at higher concentrations than in tertiary effluents. Monitoring at this stage can help evaluate treatment performance and inform upstream control strategies. Because secondary effluents and biosolids are often discharged to receiving waters or reused in agriculture without further treatment, these matrices are particularly relevant targets for surveillance.

When comparing concentration methods, the AP approach consistently yielded higher ARG concentrations than FC, by about 0.8 log units. FC values in our study were similar to previous reports with filter-based methods [31,37], despite differences in pore size, and earlier work has also noted potential clogging issues in comparable matrices [32]. The enhanced recovery with AP may reflect better retention of particulates and extracellular DNA or nucleic acid aggregation facilitated by aluminum salts [19,47,48]. In contrast, FC requires multiple handling steps that may contribute to material loss. From a practical standpoint, differences above 0.5–1 log unit can be operationally significant, particularly when ARG concentrations are near detection limits. In low-abundance scenarios, such as advanced treatment effluents or phage-associated fractions, underestimation may lead to false negatives or risk misclassification. Our findings do not imply that higher quantities automatically indicate a superior method, but that recovery differences affect sensitivity, comparability, and false-negative risk. Considering these aspects, together with higher material costs, AP appears more suitable than FC for routine monitoring.

The comparison between qPCR and ddPCR also revealed consistent differences, especially in secondary effluent. With AP concentrates, ddPCR detected higher levels of all targets than qPCR, often exceeding 1 log unit, consistent with reports of ddPCR’s greater sensitivity and tolerance to inhibitors in complex matrices [49,50]. Unlike qPCR, ddPCR enables absolute quantification without standard curves and is better suited to low-abundance or degraded DNA. These features likely contributed to its improved performance in wastewater. Prior studies also noted enhanced ARG detection with ddPCR, though few directly compared it to qPCR [51,52]. Park et al. [50] concluded that both techniques have value depending on context: qPCR offers broader dynamic range, cost-efficiency, and suitability for targeted screening, whereas ddPCR provides superior sensitivity but requires more optimization. Although this study did not include internal controls such as spiked standards to estimate absolute recovery efficiency, all methods were applied in parallel to identical aliquots, minimizing heterogeneity and enabling robust relative comparisons. Future work should incorporate internal standards to strengthen method validation and cross-laboratory comparability.

Beyond confirming previous observations, this study extends comparative analyses of qPCR and ddPCR by including both total and phage-associated ARGs in secondary treated wastewater and biosolids. Cave et al. [49] reported that ddPCR outperformed qPCR for low-copy genes such as sul1 and *qnrB* in organic-rich samples, with detection limits of ~1.6 copies for ddPCR vs. 15 for qPCR. Similarly, Di Cesare et al. [53] found ddPCR more sensitive than qPCR for sul2 and *intI1* in marine waters, detecting these genes in 48–76% of samples vs. 21–52% with qPCR. These findings support ddPCR as a robust tool for ARG quantification in complex matrices, as also observed in our study. Our results further indicate that ddPCR can serve as a highly sensitive option for ARG detection in secondary wastewater, consistent with prior reports [54]. Other approaches have also been applied, for example, Giron-Guzmán et al. [55] used high-throughput qPCR (HT-qPCR) with AP to detect multiple ARGs. Although their gene panel differed, it targeted the same antibiotic classes. Interestingly, no tetracycline- or quinolone-associated ARGs were detected, whereas β-lactam and phenicol genes were consistently found at ~6.3–6.6 log gc/mL, values similar to the *bla_CTX-M_ group 1* and *catI* concentrations observed here, reinforcing their prevalence in treated wastewater and biosolids.

In biosolids, qPCR consistently yielded higher ARG concentrations than ddPCR. Although ddPCR is often considered more tolerant to inhibitors [56,57], its performance can be compromised in complex matrices with high organic content. Uneven inhibitor distribution across droplets may reduce amplification efficiency, whereas qPCR amplifies the full volume and may buffer localized effects. Supporting this, a 1000-fold dilution of biosolids increased ddPCR signals by up to 4 log units, indicating that inhibition limited detection in undiluted samples. Similar improvements have been reported when dilutions or additives such as BSA are used to mitigate inhibition [58]. These observations align with the EMMI guidelines proposed by Borchardt et al. [59], which emphasize assessing and reporting inhibition and incorporating controls such as internal standards or spike-in assays to improve cross-laboratory comparability. While partitioning in ddPCR can help mitigate inhibition [49,60,61], the method can also suffer from droplet saturation at high target concentrations, limiting its dynamic range compared to qPCR [62,63]. This is consistent with our findings, where qPCR detected 16S rRNA above 8 log gc/g in biosolids, suggesting ddPCR saturation. Similar effects have been reported in milk and other complex samples, where ddPCR quantification failed above 5 log gc/reaction unless diluted [64,65]. In our study, diluting biosolids markedly improved ddPCR detection of 16S rRNA and *tet(A)*, highlighting the importance of ensuring that target DNA concentrations fall within ddPCR’s dynamic range.

The concentration ranges obtained in this work are consistent with those reported in previous studies evaluating ARGs in treated wastewater and biosolids. Comparable levels, typically between 10^2^ and 10^5^ gene copies/mL, have been described for *bla_CTX-M_*, *tet(A)*, and *qnrB* genes, although absolute values vary depending on the matrix type, concentration method, and quantification platform used. Similar persistence patterns of β-lactam and tetracycline resistance genes have been observed in effluents and biosolids from WWTPs worldwide [66,67,68,69]. These consistencies support the reliability of our findings and place them within the environmental ranges commonly reported in the literature.

This study investigated the presence of ARGs in the phage fraction, supporting its inclusion in AMR surveillance. Bacteriophages are more resistant than vegetative bacteria to disinfection processes such as chlorination and UV, allowing them to persist after wastewater treatment [48]. WWTPs are recognized as key hotspots for ARG dissemination due to the coexistence of antibiotic residues, resistant bacteria, and mobile genetic elements [70]. The interaction between these factors, particularly the role of phages, contributes to the environmental persistence and potential spread of resistance [71,72]. Several studies have shown that phages can retain and transfer ARGs following wastewater treatment, reinforcing the need to include phage-associated resistance in AMR surveillance efforts [27,73,74,75].

Our results are consistent with earlier reports. Colomer et al. [34,66], identified β-lactam genes, particularly *bla_CTX-M_ group 1*, as the most prevalent in the phage fraction of treated wastewater from Barcelona and Tunisia, aligning with our detection of *bla_CTX-M_ group 1* and *tet(A*). Similarly, Marti et al. [67] and Roshini et al. [69] observed β-lactam and fluoroquinolone genes, some exclusively in phages. Although studies applying ddPCR to phage fractions remain limited, De la Cruz Barron et al. [68] reported sul1 and intI1 at ~4 log gc/mL, comparable to our findings. In our study, ddPCR detected ARGs in phage-associated fractions at concentrations up to two log units higher than qPCR, confirming the presence of ARGs in DNA recovered from these fractions. While this observation indicates that resistance determinants can be associated with phage particles, it should not be interpreted as evidence that ARGs are encoded within phage genomes. Such signals may result from generalized transduction, adsorption of extracellular DNA, or co-purified bacterial DNA. Considering their persistence and resistance to conventional disinfection, bacteriophages may contribute to the environmental maintenance and mobilization of ARGs. However, the role of phages as ARG carriers remains controversial, as recent studies have reported contrasting findings regarding the frequency and mechanisms of ARG transfer through phage-mediated pathways [22,23,24,76]. Despite this, phage-associated ARGs remain understudied. By comparing detection techniques in purified phage fractions from wastewater and biosolids, our study provides novel insights and highlights the importance of incorporating phages into AMR monitoring frameworks, as well as the need for further investigation into their role in resistance dissemination.

Although this study focused on four ARGs and a limited set of samples from a single date, the use of parallel processing and technical replicates allowed us to identify consistent trends across matrices and methods. Our contribution lies not in the detection of ARGs by PCR, which has been widely applied for decades, but in the systematic comparison of concentration and detection methods under realistic conditions, and in the inclusion of the phage-associated fraction, which remains underexplored. Future studies with broader sampling schemes are needed to validate the generalizability of these findings and strengthen surveillance frameworks. Together, these findings emphasize the importance of selecting context-appropriate methods when assessing ARGs in complex matrices such as wastewater and biosolids. Aluminum-based precipitation generally improved ARG recovery compared to filtration–centrifugation in wastewater, while ddPCR enhanced sensitivity for low-abundance and phage-associated targets, and qPCR performed more consistently in biosolids, where inhibition likely limited ddPCR. These results show that methodological performance is strongly influenced by matrix and analytical context, underscoring the need for tailored approaches in AMR monitoring.

The consistent detection of ARGs in the phage fraction supports their inclusion in surveillance frameworks, given the persistence of phages and their potential role in ARG dissemination. Although limited in scope, our results contribute to refining analytical strategies and highlight the value of continued research into phage-mediated resistance and its public health implications.

Finally, although metagenomic analysis was beyond the scope of this work, integrating sequencing-based approaches in future studies would help contextualize ARG diversity, genetic backgrounds, and host associations, providing complementary validation to the quantitative data presented here. This study provides practical insights for designing more robust and reproducible monitoring strategies. The superior recovery observed with AP, particularly in phage fractions, suggests it may be advantageous when the goal is to capture low-abundance or phage-bound ARGs. Similarly, the improved sensitivity of ddPCR supports its use in complex matrices where inhibitors are present. While the study does not propose a universal protocol, it highlights methodological combinations that may guide laboratories depending on sample type and objectives. These comparative data can inform methodological choices and contribute to future discussions on the harmonization of workflows for ARG surveillance in water reuse and environmental monitoring contexts.

## 5. Conclusions

This study highlights the importance of selecting context-appropriate concentration and detection methods for quantifying ARGs in treated wastewater and biosolids. Our comparative approach demonstrated that the aluminum-based precipitation method generally provided higher recovery of ARGs than filtration–centrifugation in wastewater samples, likely due to improved retention of particulates, extracellular DNA, and bacteriophages. In terms of detection, ddPCR showed higher sensitivity than qPCR in wastewater, particularly for low-abundance targets and phage-associated ARGs, while qPCR performed more consistently in biosolids, likely due to matrix-related inhibition. These results indicate that methodological performance is strongly influenced by sample type and analytical context, suggesting that a tailored approach may be more appropriate than a universal protocol for robust environmental monitoring. Given that secondary treated wastewater and biosolids are often discharged into receiving waters or reused in agriculture without further treatment, their monitoring is essential to assess potential risks of ARG dissemination. The detection of ARGs in the phage fraction supports their inclusion in AMR surveillance frameworks, given their resistance to conventional treatment and their potential role in ARG dissemination. Together, these findings provide comparative insights that can inform methodological choices and contribute to refining analytical strategies for AMR monitoring in environmental systems, while also underscoring the value of continued research on phage-mediated gene transfer and its public health implications.

## Figures and Tables

**Table 1:** Primers, probes and cycling conditions for qPCR and ddPCR assays.

Antibiotic Group	Target Gene	Oligonucleotide	Sequence	qPCR and ddPCR Conditions	References
Tetracycline	*tet(A)*	FW	CCGCGCTTTGGGTCATT	95 °C for 10 min; 40 cycles of (95 °C for 30 s, 56 °C for 1 min)	[39]
		R	TGGTCGCGTCCCAGTGA		
		Probe	FAM-TCGGCGAGGATCG-BHQ1		
β-lactam	*bla_CTX-M_* *group 1*	FW	TTAGGAARTGTGCCGCTGYA	50 °C for 2 min; 95 °C for 3 min; 40 cycles of (95 °C for 5 s, 60 °C for 30 s, 72 °C for 1 min)	[40]
		R	CGATATCGTTGGTGGTRCCAT		
Quinolones	*qnrB*	FW	GATCGTGAAAGCCAGAAAGG	50 °C for 2 min; 95 °C for 3 min; 40 cycles of (95 °C for 5 s, 50 °C for 30 s, 72 °C for 1 min)	[41]
		R	ATGAGCAACGATGCCTGGTA		
Chloramphenicol	*catI*	FW	GGTGATATGGGATAGTGTT	50 °C for 2 min; 95 °C for 3 min; 40 cycles of (95 °C for 5 s, 55 °C for 30 s, 72 °C for 1 min)	[42]
		R	CCATCACATACTGCATGATG		
16S rRNA gene		F1048	GTGSTGCAYGGYTGTCGTCA	50 °C for 2 min; 95 °C for 3 min; 35 cycles of (95 °C for 5 s, 60 °C for 30 s, 72 °C for 1 min)	[43]
		R1194	ACGTCRTCCMCACCTTCCTC		

## Data Availability

The data presented in this study are available on request from the corresponding author.

## Supplementary Materials

The following supporting information can be downloaded at: https://www.mdpi.com/article/10.3390/pathogens14101050/s1, Figure S1. qPCR standard curves for ARGs; Figure S2. Levels of ARGs using two different detection methods (qPCR and ddPCR) for total bacteria (16S rRNA gene) and specific ARGs (tet(A), *bla*CTX-M-G1, *qnr*B and *cat*l) in secondary treated wastewater samples processed both filtration-centrifugation (FC) and aluminium-based adsorption precipitation (*AP)*; Table S1. Parameters of standard curves for ARGs: Constants *a* and *b* and their correlation coefficient (R²) for y = ax + b formula.

## References

[1] WHO Antimicrobial Resistance. https://www.who.int/news-room/fact-sheets/detail/antimicrobial-resistance.

[2] Shin H., Kim Y., Han S., Hur H.-G. (2023). Resistome Study in Aquatic Environments. J. Microbiol. Biotechnol..

[3] Drane K., Sheehan M., Whelan A., Ariel E., Kinobe R. (2024). The Role of Wastewater Treatment Plants in Dissemination of Antibiotic Resistance: Source, Measurement, Removal and Risk Assessment. Antibiotics.

[4] European Commission Regulation (EU) 2020/741 of the European Parliament and of the Council of 25 May 2020 on Minimum Requirements for Water Reuse. https://eur-lex.europa.eu/legal-content/EN/TXT/HTML/?uri=CELEX%3A32020R0741.

[5] Lin H., Li R., Chen Y., Cheng Y., Yuan Q., Luo Y. (2024). Enhanced sensitivity of extracellular antibiotic resistance genes (ARGs) to environmental concentrations of antibiotic. Chemosphere.

[6] Taylor W., Bohm K., Dyet K., Weaver L., Pattis I. (2025). Comparative analysis of qPCR and metagenomics for detecting antimicrobial resistance in wastewater: A case study. BMC Res. Notes.

[7] Liguori K., Keenum I., Calarco J., Davis B.C., Milligan E.M., Harwood V.J., Pruden A. Standardizing Methods with QA/QC Standards for Investigating the Occurrence and Removal of Antibiotic Resistant Bacteria/Antibiotic Resistance Genes (ARB/ARGs) in Surface Water, Wastewater, and Recycled Water. https://www.waterrf.org/research/projects/standardizing-methods-qaqc-standards-investigating-occurrence-and-removal.

[8] Ma C., Zhou F., Lu D., Xu S., Luo J., Gan H., Gao D., Yao Z., He W., Kurup P.U. (2024). Quantification and cultivation of *Helicobacter pylori* (*H. pylori*) from various urban water environments: A comprehensive analysis of precondition methods and sample characteristics. Environ. Int..

[9] Miller S., Greenwald H., Kennedy L.C., Kantor R.S., Jiang R., Pisarenko A., Chen E., Nelson K.L. (2022). Microbial Water Quality through a Full-Scale Advanced Wastewater Treatment Demonstration Facility. ACS ES T Eng..

[10] Tian Y., Han Z., Su D., Luan X., Yu L., Tian Z., Zhang Y., Yang M. (2024). Assessing impacts of municipal wastewater treatment plant upgrades on bacterial hazard contributions to the receiving urban river using SourceTracker. Environ. Pollut..

[11] Cheng Z.-H., Luo X.-Y., Liu D.-F., Han J., Wang H.-D., Min D., Yu H.-Q. (2024). Optimized Antibiotic Resistance Genes Monitoring Scenarios Promote Sustainability of Urban Water Cycle. Environ. Sci. Technol..

[12] Czekalski N., Gascón Díez E., Bürgmann H. (2014). Wastewater as a point source of antibiotic-resistance genes in the sediment of a freshwater lake. ISME J..

[13] van Belkum A., Burnham C.-A.D., Rossen J.W.A., Mallard F., Rochas O., Dunne W.M. (2020). Innovative and rapid antimicrobial susceptibility testing systems. Nat. Rev. Microbiol..

[14] Sun X., Wang X., Han Q., Yu Q., Wanyan R., Li H. (2024). Bibliometric analysis of papers on antibiotic resistance genes in aquatic environments on a global scale from 2012 to 2022: Evidence from universality, development and harmfulness. Sci. Total Environ..

[15] Hindson B.J., Ness K.D., Masquelier D.A., Belgrader P., Heredia N.J., Makarewicz A.J., Bright I.J., Lucero M.Y., Hiddessen A.L., Legler T.C. (2011). High-Throughput Droplet Digital PCR System for Absolute Quantitation of DNA Copy Number. Anal. Chem..

[16] Hughesman C.B., Lu X.J.D., Liu K.Y.P., Zhu Y., Poh C.F., Haynes C. (2016). A Robust Protocol for Using Multiplexed Droplet Digital PCR to Quantify Somatic Copy Number Alterations in Clinical Tissue Specimens. PLoS ONE.

[17] Taylor S.C., Laperriere G., Germain H. (2017). Droplet Digital PCR versus qPCR for gene expression analysis with low abundant targets: From variable nonsense to publication quality data. Sci. Rep..

[18] Andersson T., Adell A.D., Moreno-Switt A.I., Spégel P., Turner C., Overballe-Petersen S., Fuursted K., Lood R. (2022). Biogeographical variation in antimicrobial resistance in rivers is influenced by agriculture and is spread through bacteriophages. Environ. Microbiol..

[19] Ginn O., Nichols D., Rocha-Melogno L., Bivins A., Berendes D., Soria F., Andrade M., Deshusses M.A., Bergin M., Brown J. (2021). Antimicrobial resistance genes are enriched in aerosols near impacted urban surface waters in La Paz, Bolivia. Environ. Res..

[20] Muniesa M., García A., Miró E., Mirelis B., Prats G., Jofre J., Navarro F. (2004). Bacteriophages and Diffusion of β-lactamase Genes. Emerg. Infect. Dis..

[21] Brabban A.D., Hite E., Callaway T.R. (2005). Evolution of Foodborne Pathogens via Temperate Bacteriophage-Mediated Gene Transfer. Foodborne Pathog. Dis..

[22] Chang T.H., Pourtois J.D., Haddock N.L., Furukawa D., Kelly K.E., Amanatullah D.F., Burgener E., Milla C., Banaei N., Bollyky P.L. (2025). Prophages are infrequently associated with antibiotic resistance in Pseudomonas aeruginosa clinical isolates. mSphere.

[23] Enault F., Briet A., Bouteille L., Roux S., Sullivan M.B., Petit M.-A. (2017). Phages rarely encode antibiotic resistance genes: A cautionary tale for virome analyses. ISME J..

[24] Balcázar J.L. (2020). Implications of bacteriophages on the acquisition and spread of antibiotic resistance in the environment. Int. Microbiol..

[25] Wang Q., Wang M., Yang Q., Feng L., Zhang H., Wang R., Wang R. (2025). The role of bacteriophages in facilitating the horizontal transfer of antibiotic resistance genes in municipal wastewater treatment plants. Water Res..

[26] Brown-Jaque M., Calero-Cáceres W., Muniesa M. (2015). Transfer of antibiotic-resistance genes via phage-related mobile elements. Plasmid.

[27] Kenzaka T., Tani K., Nasu M. (2010). High-frequency phage-mediated gene transfer in freshwater environments determined at single-cell level. ISME J..

[28] Jończyk E., Kłak M., Międzybrodzki R., Górski A. (2011). The influence of external factors on bacteriophages—review. Folia Microbiol.

[29] Koutsoumanis K., Allende A., Álvarez-Ordóñez A., Bolton D., Bover-Cid S., Chemaly M., Davies R., De Cesare A., Herman L., EFSA Panel on Biological Hazards (BIOHAZ) (2021). Role played by the environment in the emergence and spread of antimicrobial resistance (AMR) through the food chain. EFSA J..

[30] Anyanwu M.U., Nwobi O.C., Okpala C.O.R., Ezeonu I.M. (2022). Mobile Tigecycline Resistance: An Emerging Health Catastrophe Requiring Urgent One Health Global Intervention. Front. Microbiol..

[31] Marathe N.P., Svanevik C.S., Ghavidel F.Z., Grevskott D.H. (2021). First report of mobile tigecycline resistance gene *tet*(X4)-harbouring multidrug-resistant *Escherichia coli* from wastewater in Norway. J. Glob. Antimicrob. Resist..

[32] Caltagirone M., Nucleo E., Spalla M., Zara F., Novazzi F., Marchetti V.M., Piazza A., Bitar I., De Cicco M., Paolucci S. (2017). Occurrence of Extended Spectrum β-Lactamases, KPC-Type, and MCR-1.2-Producing Enterobacteriaceae from Wells, River Water, and Wastewater Treatment Plants in Oltrepò Pavese Area, Northern Italy. Front. Microbiol..

[33] Amato M., Dasí D., González A., Ferrús M.A., Castillo M.Á. (2021). Occurrence of antibiotic resistant bacteria and resistance genes in agricultural irrigation waters from Valencia city (Spain). Agric. Water Manag..

[34] Colomer-Lluch M., Calero-Cáceres W., Jebri S., Hmaied F., Muniesa M., Jofre J. (2014). Antibiotic resistance genes in bacterial and bacteriophage fractions of Tunisian and Spanish wastewaters as markers to compare the antibiotic resistance patterns in each population. Environ. Int..

[35] Keenum I., Liguori K., Calarco J., Davis B.C., Milligan E., Harwood V.J., Pruden A. (2022). A framework for standardized qPCR-targets and protocols for quantifying antibiotic resistance in surface water, recycled water and wastewater. Crit. Rev. Environ. Sci. Technol..

[36] Oliveira M., Truchado P., Cordero-García R., Gil M.I., Soler M.A., Rancaño A., García F., Álvarez-Ordóñez A., Allende A. (2023). Surveillance on ESBL-Escherichia coli and Indicator ARG in Wastewater and Reclaimed Water of Four Regions of Spain: Impact of Different Disinfection Treatments. Antibiotics.

[37] Pérez-Cataluña A., Cuevas-Ferrando E., Randazzo W., Falcó I., Allende A., Sánchez G. (2021). Comparing analytical methods to detect SARS-CoV-2 in wastewater. Sci. Total Environ..

[38] Larrañaga O., Brown-Jaque M., Quirós P., Gómez-Gómez C., Blanch A.R., Rodríguez-Rubio L., Muniesa M. (2018). Phage particles harboring antibiotic resistance genes in fresh-cut vegetables and agricultural soil. Environ. Int..

[39] Khan S.A., Sung K., Nawaz M.S. (2011). Detection of aacA-aphD, qacEδ1, marA, floR, and tetA genes from multidrug-resistant bacteria: Comparative analysis of real-time multiplex PCR assays using EvaGreen^®^ and SYBR^®^ Green I dyes. Mol. Cell. Probes.

[40] Dallenne C., Da Costa A., Decré D., Favier C., Arlet G. (2010). Development of a set of multiplex PCR assays for the detection of genes encoding important β-lactamases in Enterobacteriaceae. J. Antimicrob. Chemother..

[41] Kim H.B., Park C.H., Kim C.J., Kim E.-C., Jacoby G.A., Hooper D.C. (2009). Prevalence of Plasmid-Mediated Quinolone Resistance Determinants over a 9-Year Period. Antimicrob. Agents Chemother..

[42] Yoo M.H., Huh M.-D., Kim E., Lee H.-H., Jeong H.D. (2003). Characterization of chloramphenicol acetyltransferase gene by multiplex polymerase chain reaction in multidrug-resistant strains isolated from aquatic environments. Aquaculture.

[43] Maeda H., Fujimoto C., Haruki Y., Maeda T., Kokeguchi S., Petelin M., Arai H., Tanimoto I., Nishimura F., Takashiba S. (2003). Quantitative real-time PCR using TaqMan and SYBR Green for Actinobacillus actinomycetemcomitans, Porphyromonas gingivalis, Prevotella intermedia, tetQ gene and total bacteria. FEMS Immunol. Med. Microbiol..

[44] Bonanno Ferraro G., Bonomo C., Brandtner D., Mancini P., Veneri C., Briancesco R., Coccia A.M., Lucentini L., Suffredini E., Bongiorno D. (2024). Characterisation of microbial communities and quantification of antibiotic resistance genes in Italian wastewater treatment plants using 16S rRNA sequencing and digital PCR. Sci. Tot. Environ..

[45] Ejaz H., Younas S., Abosalif K.O.A., Junaid K., Alzahrani B., Alsrhani A., Abdalla A.E., Ullah M.I., Qamar M.U., Hamam S.S.M. (2021). Molecular analysis of blaSHV, blaTEM, and blaCTX-M in extended-spectrum β-lactamase producing Enterobacteriaceae recovered from fecal specimens of animals. PLoS ONE.

[46] Sghaier S., Abbassi M.S., Pascual A., Serrano L., Díaz-De-Alba P., Said M.B., Hassen B., Ibrahim C., Hassen A., López-Cerero L. (2019). Extended-spectrum β-lactamase-producing Enterobacteriaceae from animal origin and wastewater in Tunisia: First detection of O25b-B23-CTX-M-27-ST131 *Escherichia coli* and CTX-M-15/OXA-204-producing *Citrobacter freundii* from wastewater. J. Glob. Antimicrob. Resist..

[47] Nordgren J., Matussek A., Mattsson A., Svensson L., Lindgren P.-E. (2009). Prevalence of norovirus and factors influencing virus concentrations during one year in a full-scale wastewater treatment plant. Water Res..

[48] Calero-Cáceres W., Muniesa M. (2016). Persistence of naturally occurring antibiotic resistance genes in the bacteria and bacteriophage fractions of wastewater. Water Res..

[49] Cavé L., Brothier E., Abrouk D., Bouda P.S., Hien E., Nazaret S. (2016). Efficiency and sensitivity of the digital droplet PCR for the quantification of antibiotic resistance genes in soils and organic residues. Appl. Microbiol. Biotechnol..

[50] Park S., Rana A., Sung W., Munir M. (2021). Competitiveness of Quantitative Polymerase Chain Reaction (qPCR) and Droplet Digital Polymerase Chain Reaction (ddPCR) Technologies, with a Particular Focus on Detection of Antibiotic Resistance Genes (ARGs). Appl. Microbiol..

[51] Baraka V., Andersson T., Makenga G., Francis F., Minja D.T.R., Overballe-Petersen S., Tang M.-H.E., Fuursted K., Lood R. (2023). Unveiling Rare Pathogens and Antibiotic Resistance in Tanzanian Cholera Outbreak Waters. Microorganisms.

[52] Leão I., Khalifa L., Gallois N., Vaz-Moreira I., Klümper U., Youdkes D., Palmony S., Dagai L., Berendonk T.U., Merlin C. (2023). Microbiome and Resistome Profiles along a Sewage-Effluent-Reservoir Trajectory Underline the Role of Natural Attenuation in Wastewater Stabilization Reservoirs. Appl. Environ. Microbiol..

[53] Di Cesare A., Petrin S., Fontaneto D., Losasso C., Eckert E.M., Tassistro G., Borello A., Ricci A., Wilson W.H., Pruzzo C. (2018). ddPCR applied on archived Continuous Plankton Recorder samples reveals long-term occurrence of class 1 integrons and a sulphonamide resistance gene in marine plankton communities. Environ. Microbiol. Rep..

[54] Zieliński W., Korzeniewska E., Harnisz M., Drzymała J., Felis E., Bajkacz S. (2021). Wastewater treatment plants as a reservoir of integrase and antibiotic resistance genes—An epidemiological threat to workers and environment. Environ. Int..

[55] Girón-Guzmán I., Sánchez-Alberola S., Cuevas-Ferrando E., Falcó I., Díaz-Reolid A., Puchades-Colera P., Ballesteros S., Pérez-Cataluña A., Coll J.M., Núñez E. (2024). Longitudinal study on the multifactorial public health risks associated with sewage reclamation. npj Clean Water.

[56] Dingle T.C., Sedlak R.H., Cook L., Jerome K.R. (2013). Tolerance of Droplet-Digital PCR vs Real-Time Quantitative PCR to Inhibitory Substances. Clin. Chem..

[57] Sidstedt M., Rådström P., Hedman J. (2020). PCR inhibition in qPCR, dPCR and MPS—Mechanisms and solutions. Anal. Bioanal. Chem..

[58] Lawaju B.R., Yan G. (2024). Assessment of Common Factors Associated with Droplet Digital PCR (ddPCR) Quantification of Paratrichodorus allius in Soil. Int. J. Mol. Sci..

[59] Borchardt M.A., Boehm A.B., Salit M., Spencer S.K., Wigginton K.R., Noble R.T. (2021). The Environmental Microbiology Minimum Information (EMMI) Guidelines: qPCR and dPCR Quality and Reporting for Environmental Microbiology. Environ. Sci. Technol..

[60] Cristiano D., Peruzy M.F., Aponte M., Mancusi A., Proroga Y.T.R., Capuano F., Murru N. (2021). Comparison of droplet digital PCR vs real-time PCR for *Yersinia enterocolitica* detection in vegetables. Int. J. Food Microbiol..

[61] Villamil C., Calderon M.N., Arias M.M., Leguizamon J.E. (2020). Validation of Droplet Digital Polymerase Chain Reaction for Salmonella spp. Quantification. Front. Microbiol..

[62] Choi C.-H., Kim E., Yang S.-M., Kim D.-S., Suh S.-M., Lee G.-Y., Kim H.-Y. (2022). Comparison of Real-Time PCR and Droplet Digital PCR for the Quantitative Detection of *Lactiplantibacillus plantarum* subsp. *plantarum*. Foods.

[63] Wang M., Yang J., Gai Z., Huo S., Zhu J., Li J., Wang R., Xing S., Shi G., Shi F. (2018). Comparison between digital PCR and real-time PCR in detection of *Salmonella typhimurium* in milk. Int. J. Food Microbiol..

[64] Zhao Y., Xia Q., Yin Y., Wang Z. (2016). Comparison of Droplet Digital PCR and Quantitative PCR Assays for Quantitative Detection of Xanthomonas citri Subsp. citri. PLoS ONE.

[65] Porcellato D., Narvhus J., Skeie S.B. (2016). Detection and quantification of *Bacillus cereus* group in milk by droplet digital PCR. J. Microbiol. Methods.

[66] Colomer-Lluch M., Jofre J., Muniesa M. (2011). Antibiotic Resistance Genes in the Bacteriophage DNA Fraction of Environmental Samples. PLoS ONE.

[67] Marti E., Variatza E., Balcázar J.L. (2014). Bacteriophages as a reservoir of extended-spectrum β -lactamase and fluoroquinolone resistance genes in the environment. Clin. Microbiol. Infect..

[68] de la Cruz Barron M., Kneis D., Elena A.X., Bagra K., Berendonk T.U., Klümper U. (2023). Quantification of the mobility potential of antibiotic resistance genes through multiplexed ddPCR linkage analysis. FEMS Microbiol Ecol.

[69] Roshini J., Raj M., Karunasagar I. (2017). Prevalence of blaCTX-M-15 in Coliphages Isolated from Sewage. Adv. Sci. Lett..

[70] Wang J., Xu S., Zhao K., Song G., Zhao S., Liu R. (2023). Risk control of antibiotics, antibiotic resistance genes (ARGs) and antibiotic resistant bacteria (ARB) during sewage sludge treatment and disposal: A review. Sci. Total Environ..

[71] Manaia C.M., Rocha J., Scaccia N., Marano R., Radu E., Biancullo F., Cerqueira F., Fortunato G., Iakovides I.C., Zammit I. (2018). Antibiotic resistance in wastewater treatment plants: Tackling the black box. Environ. Int..

[72] Rizzo L., Manaia C., Merlin C., Schwartz T., Dagot C., Ploy M.C., Michael I., Fatta-Kassinos D. (2013). Urban wastewater treatment plants as hotspots for antibiotic resistant bacteria and genes spread into the environment: A review. Sci. Total Environ..

[73] Feng G., Huang H., Chen Y. (2021). Effects of emerging pollutants on the occurrence and transfer of antibiotic resistance genes: A review. J. Hazard. Mater..

[74] Jiang S.C., Paul J.H. (1998). Gene Transfer by Transduction in the Marine Environment. Appl. Environ. Microbiol..

[75] von Wintersdorff C.J.H., Penders J., van Niekerk J.M., Mills N.D., Majumder S., van Alphen L.B., Savelkoul P.H.M., Wolffs P.F.G. (2016). Dissemination of Antimicrobial Resistance in Microbial Ecosystems through Horizontal Gene Transfer. Front. Microbiol..

[76] Cross B.J., Partridge S.R., Sheppard A.E. (2026). Impacts of mobile genetic elements on antimicrobial resistance genes in gram-negative pathogens: Current insights and genomic approaches. Microbiol. Res..

