# Supplementary material for: Comparative Analysis of Concentration and Quantification Methods for Antibiotic Resistance Genes and Their Phage-Mediated Dissemination in Treated Wastewater and Biosolids
Source: Pathogens. 2025 Oct 18;14(10):1050. doi: 10.3390/pathogens14101050 (PMC12566704; doi:10.3390/pathogens14101050)
Supplement: Supplementary file 1 [file pathogens-14-01050-s001.zip › pathogens-3917373-supplementary.pdf]

Comparative Analysis of Concentration and Quantification Methods for Antibiotic Resistance Genes and Their Phage-Mediated Dissemination in Treated Wastewater and Biosolids

Irene Falcó [1,2,*], Ana Allende [2], Francesca Cutripi [3], Rosa Aznar [1], Gloria Sánchez [4] and Pilar Truchado [2,*]

[1] Department of Microbiology and Ecology, University of Valencia, 46100 Valencia, Spain
[2] Research Group on Microbiology and Quality of Fruits and Vegetables (MxQ), Department of Food Science and Technology, CEBAS-CSIC, Campus Universitario de Espinardo, 25, 30100 Murcia, Spain
[3] CIBIO—Department of Cellular, Computational and Integrative Biology, University of Trento, 38123 Trento, Italy
[4] Environmental Virology and Food Safety Lab (VISAFELab), Department of Preservation and Food Safety Technologies, Institute of Agrochemistry and Food Technology, IATA-CSIC, Av. Agustín Escardino 7, 46980 Valencia, Spain
* Correspondence: irene.falco@iata.csic.es (I.F.); ptruchado@cebas.csic.es (P.T.)

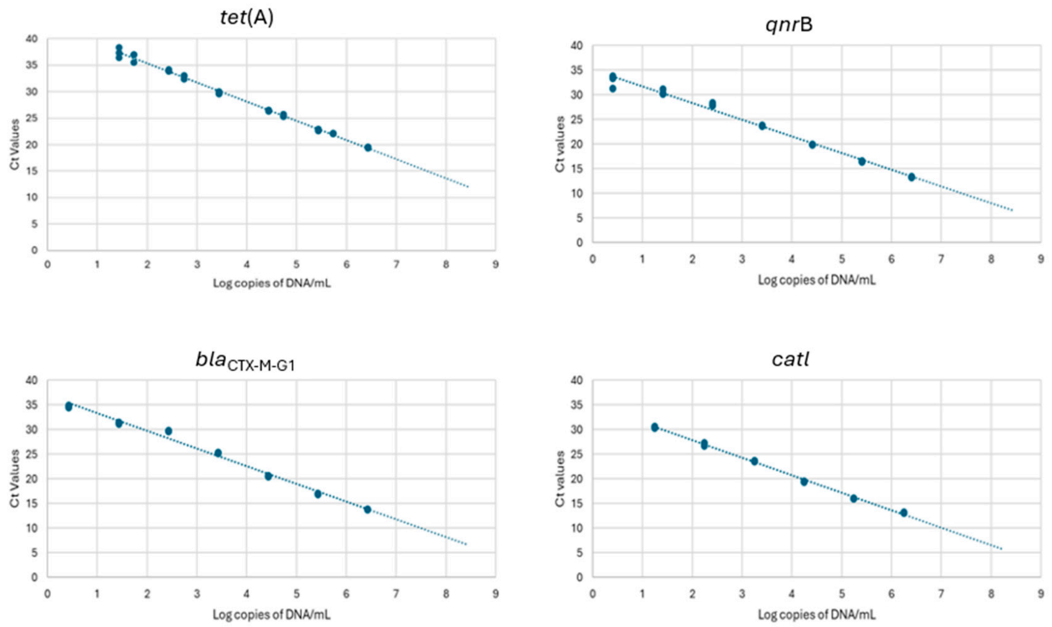

Figure S1. qPCR standard curves for ARGs.

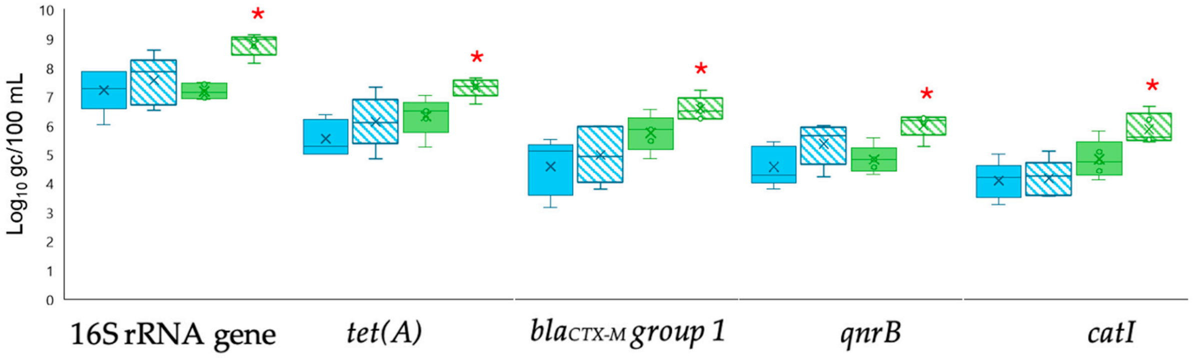

Figure S2. Levels of ARGs using two different detection methods (qPCR and ddPCR) for total bacteria (16S rRNA gene) and specific ARGs (tet(A), *bla*$_{CTX-M-G1}$, *qnr*B and *cat*I) in secondary treated wastewater samples processed both filtration-centrifugation (FC) and aluminium-based adsorption precipitation (AP). Box plots represent the interquartile interval, where 50% of the data is the median (middle quartile) and the lower and upper quartiles (25 and 75% of the scores, respectively). Solid colours indicate filtration-centrifugation protocol (FC); dashed colour*s* indicate aluminium-based adsorption precipitation protocol (AP).
* Indicates significant differences between FC and AP ($p < 0.05$).

Table S1. Parameters of standard curves for ARGs: Constants *a* and *b* and their correlation coefficient (R^2) for y = ax + b formula.

**ARG**	***a***	***b***	$\mathbf{R^2}$
***tet*(A)**	-3.6186	42.599	0.9954
$\boldsymbol{bla_{CTX\text{-}M\text{-}G1}}$	-3.5877	36.92	0.9892
***qnr*B**	-3.3882	35.069	0.9881
***cat*I**	-3.5562	34.949	0.9974
